# Evaluation of the NMP22 BladderChek test for detecting bladder cancer: a systematic review and meta-analysis

**DOI:** 10.18632/oncotarget.22065

**Published:** 2017-10-23

**Authors:** Zijie Wang, Hongliang Que, Chuanjian Suo, Zhijian Han, Jun Tao, Zhengkai Huang, Xiaobin Ju, Ruoyun Tan, Min Gu

**Affiliations:** ^1^ Department of Urology, The First Affiliated Hospital with Nanjing Medical University, Nanjing, 210029, China

**Keywords:** bladder cancer, NMP22 BladderChek test, diagnostic, systematic review, meta-analysis

## Abstract

**Background:**

We examined the usefulness of the nuclear matrix protein 22 (NMP22) BladderChek test for detecting bladder cancer.

**Materials and Methods:**

A literature search was performed using PubMed, Embase, the Cochrane Library, and Web of Science. The diagnostic accuracy of the NMP22 BladderChek test was evaluated via pooled sensitivity, specificity, positive likelihood ratio (PLR), negative likelihood ratio (NLR), diagnostic odds ratio (DOR), and area under curve (AUC). Inter-study heterogeneity was explored using meta-regression and subgroup analyses.

**Results:**

We included 23 studies in the systematic review and 19 in the quantitative meta-analysis. Overall sensitivity and specificity were 56% (52–59%) and 88% (87–89%), respectively; pooled PLR and NLR were 4.36 (3.02–6.29) and 0.51 (0.40–0.66), respectively; DOR was 9.29 (5.55–15.55) with an AUC of 0.8295. The mean sensitivity for Ta, T1, ≥ T2, Tis, G1, G2, and G3 disease was 13.68%, 29.49%, 74.03%, 34.62%, 44.16%, 56.25%, and 67.34%, respectively.

**Conclusions:**

The NMP22 BladderChek test shows good discrimination ability for detecting bladder cancer and a high-specificity algorithm that can be used for early detection to rule out patients with higher bladder cancer risk. It also has better potential for screening higher-grade and higher-stage tumors, and better diagnostic performance in Asians.

## INTRODUCTION

Bladder cancer, commonly referred to as carcinoma of the epithelial lining of the urinary bladder, is one of most common male cancers worldwide [1]. In the United States, an estimated 54,610 men were diagnosed with a new occurrence of bladder cancer in 2013; 17,960 women were diagnosed with the malignancy in the same year [2]. The probability of developing bladder cancer sharply increases with age, from 0.02% at 39 years to 3.69% at > 70 years [3]. Therefore, early diagnosis for people at higher risk of bladder cancer is crucial for prolonging the rate of survival and for increasing the quality of life.

In general, the modalities for early diagnosis and follow-up of bladder cancer include cystoscopy and urine cytology [4]. Cystoscopy is considered the gold standard for the initial diagnosis and staging of bladder cancer, which should be confirmed by histological examination of biopsy specimens [5]. In recent years, scientific and clinical research has intensified to identify non-invasive methods for predicting bladder cancer occurrence, such as microRNAs, bladder tumor antigen (BTA) stat, and UroVysion fluorescence *in situ* hybridization (FISH) [6–8]. However, these current non-invasive technologies do not accurately reflect the development and detailed information of bladder cancer, including the disease grade and stage.

Another novel assay that has been used for detecting bladder cancer is the nuclear matrix protein 22 (NMP22) test. In this test, nuclear mitotic apparatus protein 1 (NUMA1) levels are assessed using monoclonal antibodies [9]. Two assay formats, i.e., the NMP22 Bladder Cancer ELISA (enzyme-linked immunosorbent assay) Test Kit and the NMP22 BladderChek point-of-care (POC) test (Alere Scarborough, Inc., Waltham, MA, USA), have been approved by the US Food and Drug Administration (FDA) for bladder cancer detection and surveillance in urine samples [10]. Several diagnostic trials have explored the efficacy of the NMP22 BladderChek test in bladder cancer detection and follow-up in recent years. However, its diagnostic performance, especially its sensitivity and specificity, varied across these studies (Table 1), resulting in its diagnostic accuracy being unclear.

**Table 1:** Basic characteristics of eligible studies in our meta-analysis

Study	Ethnicity	Case number	Mean age (year; range)	Male/female	Proportion of smoking patients (%)	Specific details of index test used	Total sensitivity (%)	Total specificity (%)
**P.M.J. Moonen (2005)**	Caucasian	106	66.4 (26.9–86.1)	79/27	NA	NMP22BC, Cytology	63.63%	90%
**S. Tritschler (2006)**	Asian	100	67.9	71/29	NA	NMP22BC	65.00%	40
**A. Kumar (2006)**	Asian	131	67 (32–91)	117/14	NA	NMP22BC, Cytology	84.80%	77.6%
**Y. Lotan (2007)**	Caucasian	1328	58.7 (18–96)	756/572	36.1%	NMP22BC	56.96%	85.8%
**Y. Lotan (2008)**	Caucasian	1502	62.5 (46–92)	1175/327	45%	NMP22BC	NA	NA
**H. Steiner (2008)**	Caucasian	183	60.1 (36.8–83.8)	123/60	100%	Dipstick, NMP22BC, Cytology, UroVysion	5.56%	82.5%
**V K. Arora (2009)**	Asian	53	59 (33–83)	48/5	66.04%	NMP22BC, Cytology, Cystoscopy	78.95%	80
**H S. Choi (2009)**	Asian	1070	59.31	650/420	NA	NMP22BC	77.50%	88.8
**E O. Kehinde (2011)**	Asian	178	55.3 (16–77)	NA	80%	Cytology, NMP22BC, UroVysion	82.00%	66.0%
**T. Smrkolj (2011)**	Caucasian	108	68.3	74/34	NA	Cytology, NMP22BC	17.70%	100.0%
**E C. Hwang (2011)**	Asian	1021	65	776/245	NA	NMP22BC	22.58%	97.97
**L. Sagnak (2011)**	Asian	164	30.8	56/108	NA	NMP22BC	100.00%	85.2%
**M A. Maghrebi (2012)**	Asian	105	53.48 (16–77)	81/24	NA	NMP22BC	61.30%	96
**G. Hatzichristodoulou (2012)**	Caucasian	200	61.3 (48–75)	142/58	NA	NMP22 ELISA, NMP22BC	59.00%	93.0%
**G. Ludecke (2012)**	Caucasian	13	< 50	NA	NA	UBC rapid, NMP22BC, BTA stat	NA	NA
**E. Coskuner (2012)**	Asian	95	60.7 (27–88)	78/17	NA	NMP22BC	44.40%	98.4%
**P O. Sullivan (2012)**	Caucasian	475	69 (59–77)	389/96	76%	Cytology, microscopy, NMP22BC, NMP22 ELISA	37.70%	96.40%
**HX. Li (2013)**	Asian	175	62.4 (23–89)	142/33	NA	LBC, FISH, NMP22BC	67.60%	88.1%
**R. Ritter (2013)**	Caucasian	198	70 (20–90)	151/47	NA	UBC ELISA, Cytology, NMP22BC, UBC rapid	16.40%	95.3%
**F A. Yafi (2014)**	Caucasian	109	69 (33–96)	90/19	89%	dipstick, BTA Stat, NMP22 BC, ImmunoCyt	58.00%	85
**L. Turkeri (2014)**	Asian	303	56.6	146/157	NA	NMP22 BC, RisikoCheck	45.00%	95.0%
**M D. Bell (2016)**	Caucasian	91	74 (45–96)	76/15	89%	Dipstick, BTA Stat, NMP22 BC, ImmunoCyt	NA	NA
**Y. Lotan (2017)**	Caucasian	1016	20–90	786/230	NA	Cytology, NMP22 ELISA, NMP22 BC, UroVysion	11.0%	NA

Abbreviations: NMP22BC, NMP22 BladderChek; ELISA, Enzyme-linked immunosorbent assay; LBC, liquid-based cytology; FISH, fluorescence in situ hybridization; NA, not available.

Accordingly, in this systematic review and meta-analysis, we comprehensively reviewed these diagnostic trials and investigated the diagnostic value of the NMP22 BladderChek test in bladder cancer detection and follow-up according to the PRISMA (preferred reporting items for systematic reviews and meta-analysis) guidelines.

## RESULTS

### Study selection and characteristics

**Figure 1:**
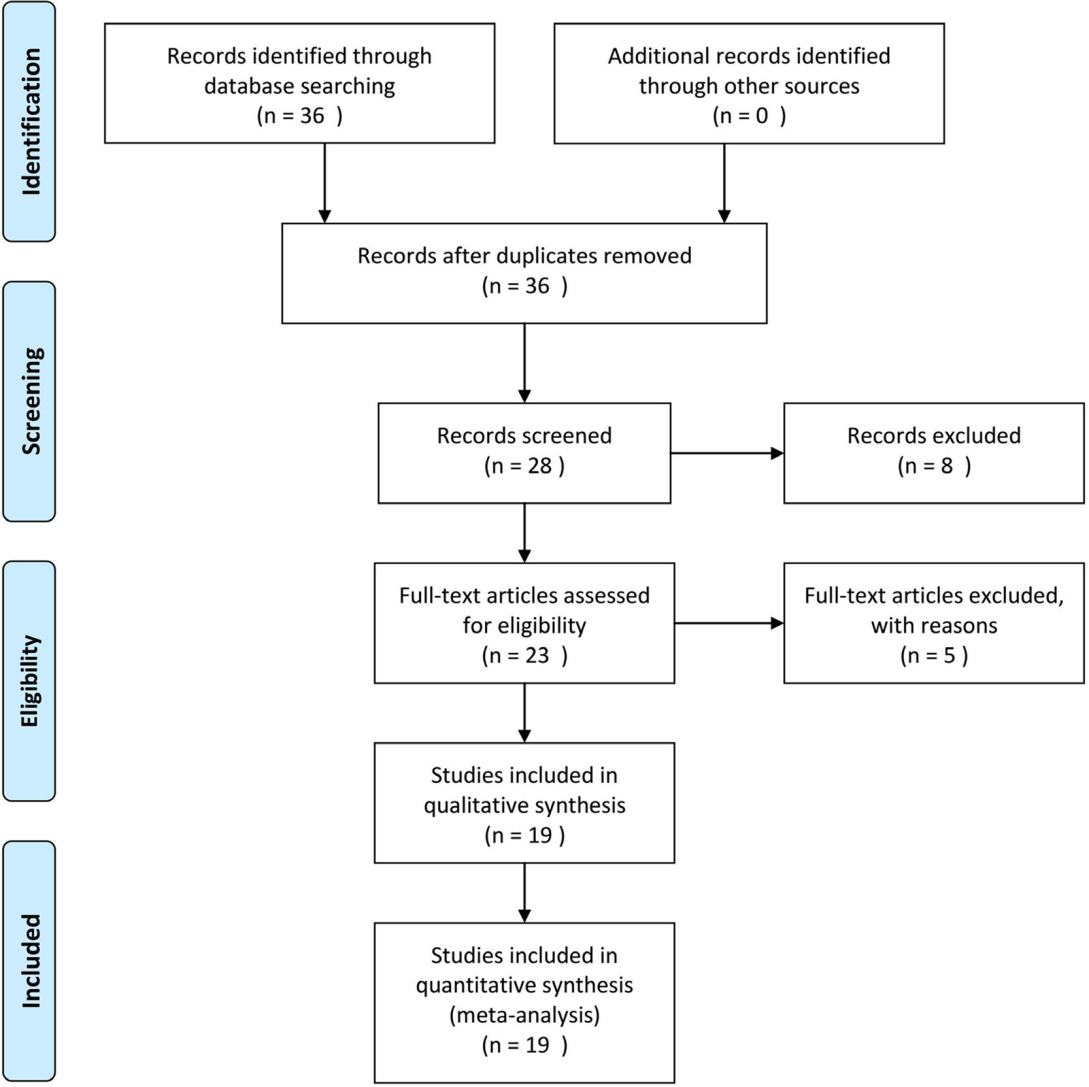
Flow chart for identification of eligible studies

Figure 1 shows the results of the literature searched and selected according to the PRISMA guidelines. A total 36 studies were identified from the primary literature review. Next, 28 studies were included for full-text review, and eight were excluded because they were reviews or were studies in languages other than English or Chinese. Subsequently, 23 studies [15–36] involving 8724 patients were considered eligible for the systematic review, and 19 studies involving 5291 patients were included in the meta-analysis.

**Figure 2:**
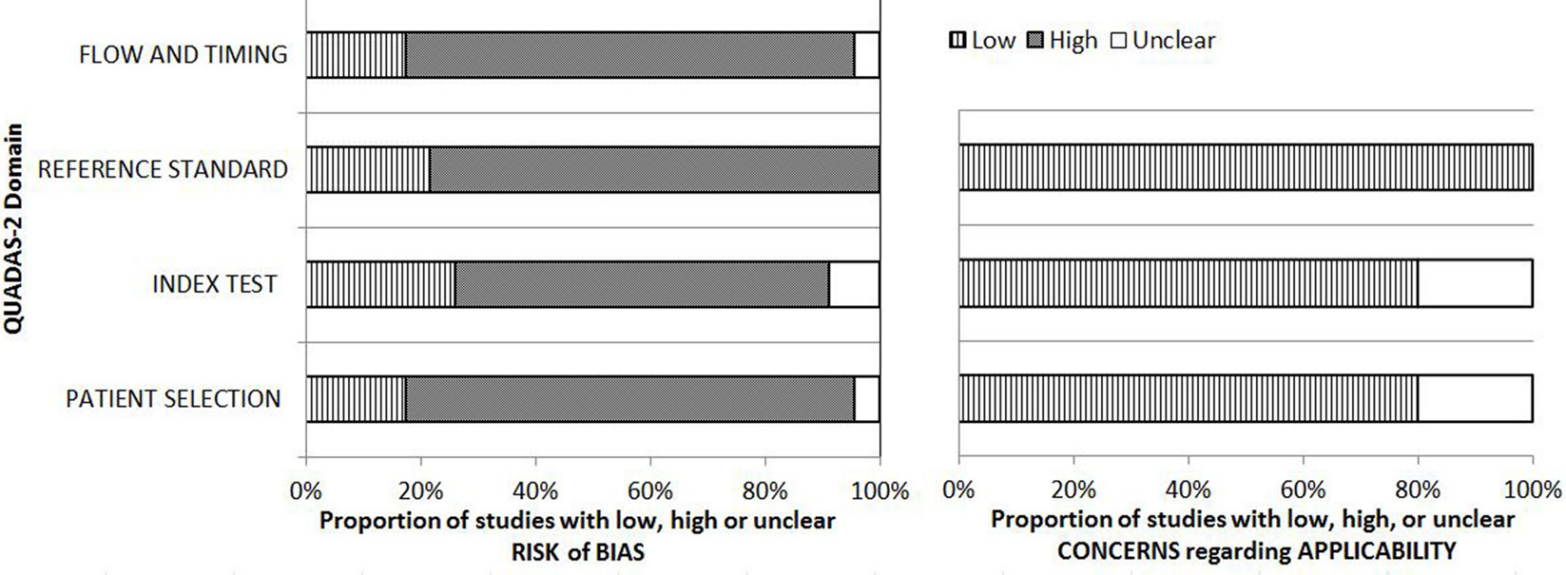
Results of QUADAS-2 quality assessment of included studies

Overall, 11 and 22 studies were performed in Asian populations and Caucasian populations, respectively. Most of the patients were men aged > 50 years. Twenty and 19 studies reported total sensitivity (mean, 52.75%) and specificity (mean, 86.37%), respectively. All studies included in the meta-analysis used cytology or cystoscopy results as the gold standard. Figure 2 shows an overview of the methodological quality results. In general, the overall quality of the eligible studies was high.

### Diagnostic performance

**Figure 3:**
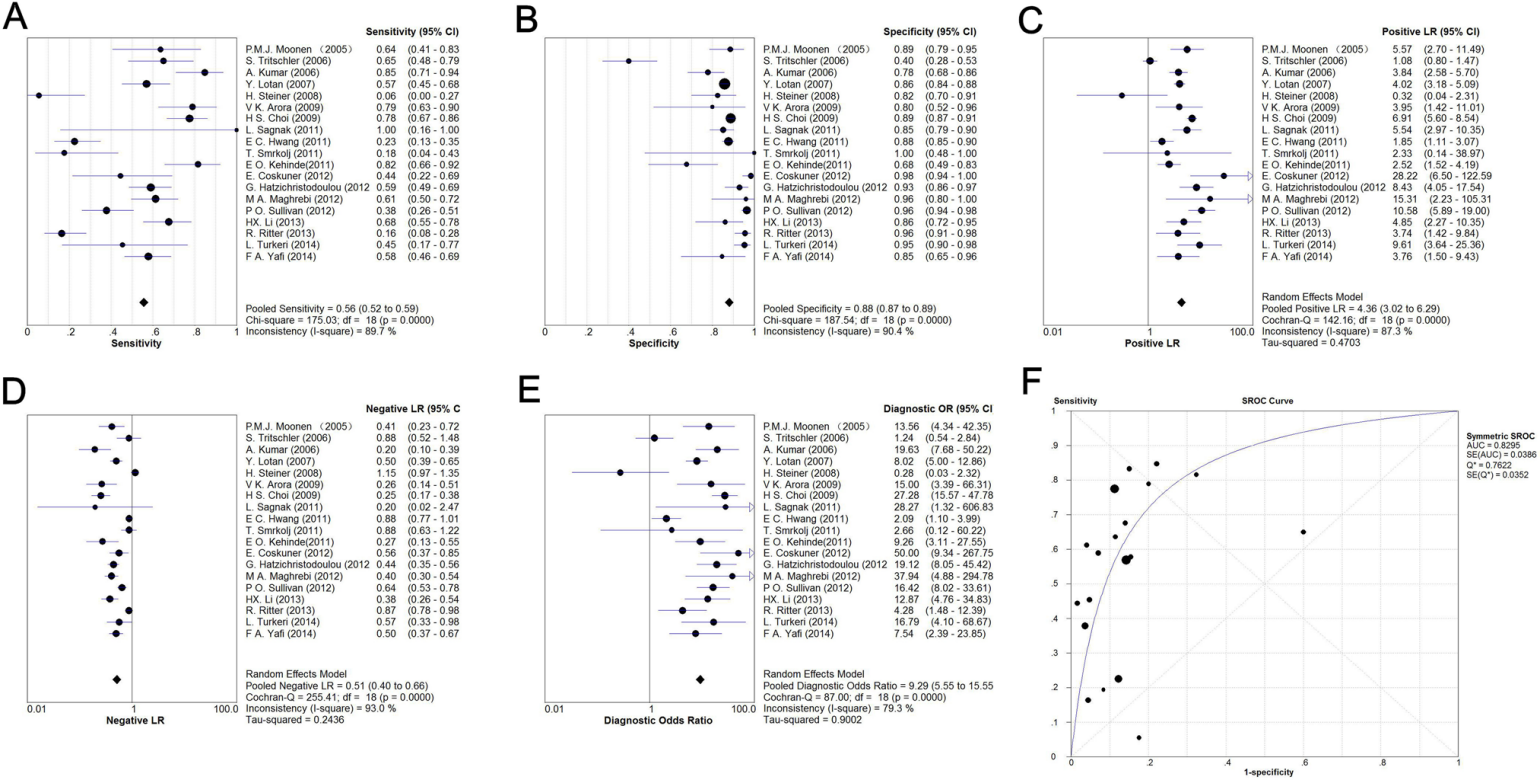
Meta-analysis of pooled sensitivity (**A**), specificity (**B**), PLR (**C**), NLR (**D**), DOR (**E**), and ROC (**F**).

Sensitivity, specificity, negative likelihood ratio (NLR), and positive likelihood ratio (PLR) values and their 95% confidence intervals (CIs) were calculated on a per-patient basis and charted on forest plots and summary receiver operating characteristic (sROC) curves (Figure 3). The overall sensitivity and specificity among the 19 studies was 56% (52–59%) and 88% (87–89%), respectively; for PLR and NLR, the pooled results were 4.36 (3.02–6.29) and 0.51 (0.40–0.66), respectively; the diagnostic odds ratio (DOR) among the studies was 9.29 (5.55–15.55) with an area under curve (AUC) of 0.83.

With respect to bladder cancer stage and grade, Supplementary Table 1 presents the sensitivity based on histological examination. The mean sensitivity for Ta, T1, ≥ T2, and Tis was 13.68%, 29.49%, 74.03%, and 34.62%, respectively; that for G1, G2, and G3 was 44.16%, 56.25%, and 67.34%, respectively.

Significant heterogeneity was observed among the 19 studies in our meta-analysis. Accordingly, we performed meta-regression analysis to explore the origins of the heterogeneity. Figure 3 shows that the heterogeneity was suggested across the studies, and among eight factors, study quality was identified as statistically significant, indicating that the quality of the studies was responsible for the relatively high heterogeneity.

To explore the effect of ethnicity and recurrence on the diagnostic value of the NMP22 BladderChek test, we performed subgroup analysis based on ethnicity and primary occurrence/recurrence; Supplementary Figure 1 and Figure 2 show the results.

### Publication bias

**Figure 4:**
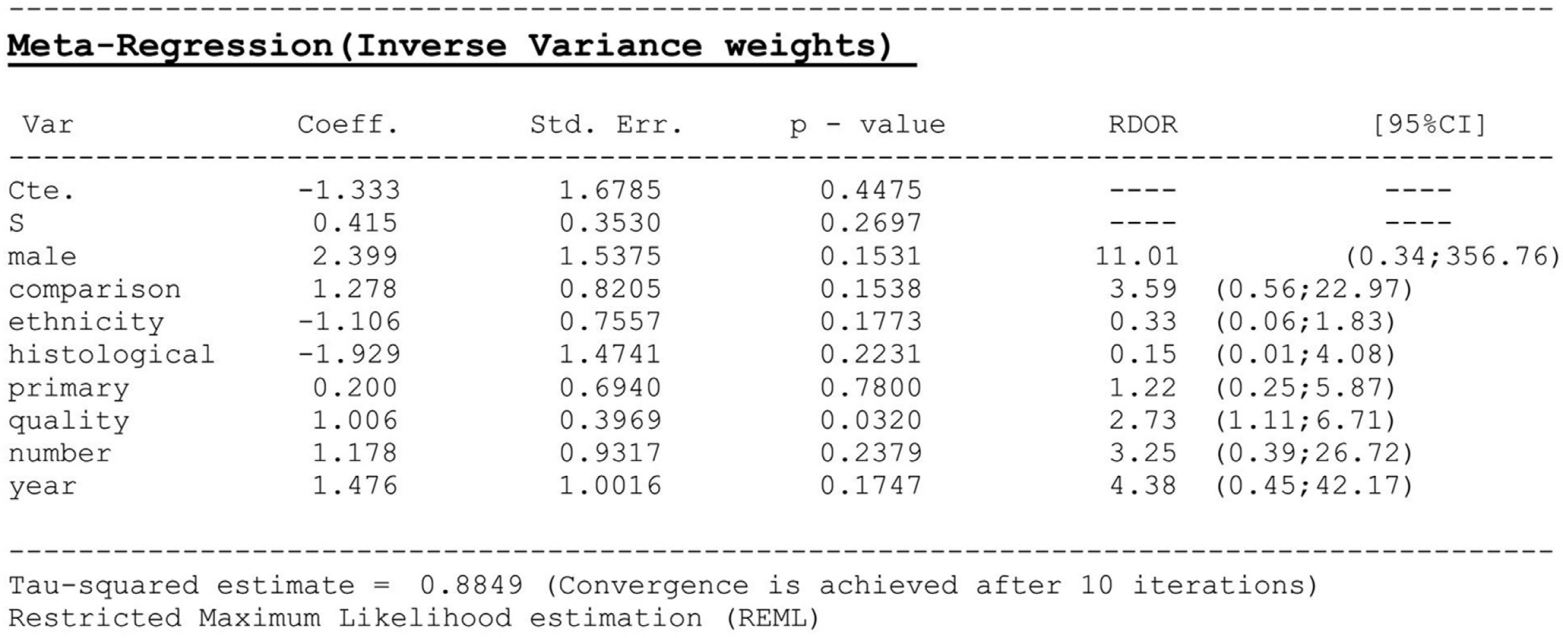
Meta-regression analysis of heterogeneity across eligible studies Male, whether the male/female ratio > 50%; comparison, whether compared with other novel diagnostic methods; ethnicity, Asian/Caucasian population; histological, whether histological examination of bladder cancer was performed; primary, whether bladder cancer recurrence was detected; quality, the quality of all included studies as assessed by QUADAS-2; number, where case number > 400; year, whether publication year was after 2011.

There was no statistically significant publication bias across the studies, the slope coefficient had a *p*-value of 0.75 (-19.11, 14.05) (Figure 4).

**Figure 5:**
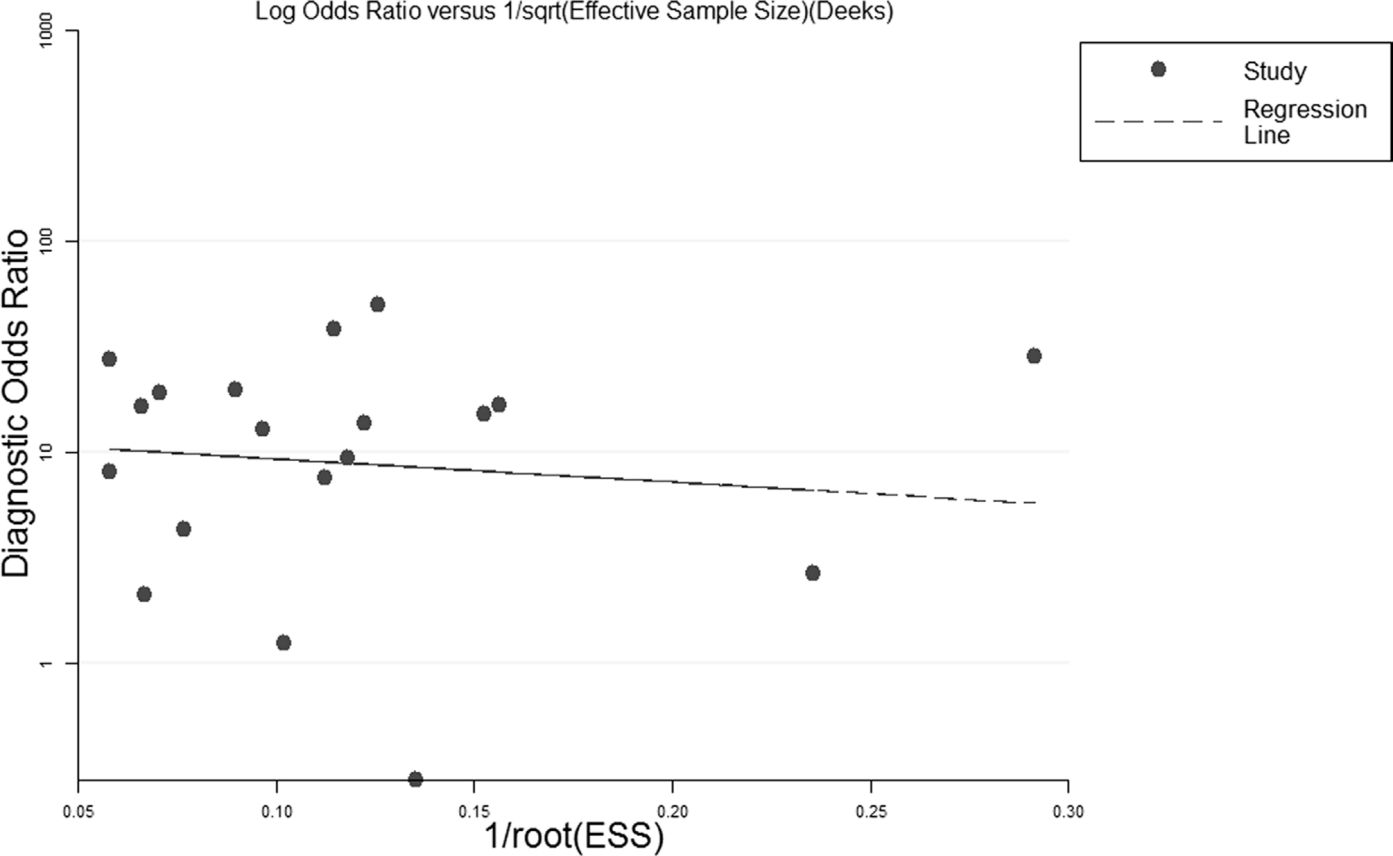
Deeks’ funnel plot asymmetry test for studies in the systematic review

## DISCUSSION

In this systematic review and meta-analysis, the NMP22 BladderChek test had pooled sensitivity of 56% and specificity of 88%, and pooled DOR of 9.29 and AUC of 0.83. Moreover, the diagnostic performance increased with disease stage and grade; the test shows good discrimination ability for detecting bladder cancer among both Asians and Caucasians. To the best of our knowledge, ours is the first systematic review and meta-analysis to assess the diagnostic value of the NMP22 BladderChek test for screening bladder cancer.

The pooled DOR was 9.29 (5.55–15.55), indicating the test’s relatively high discrimination ability. The pooled sensitivity and specificity was 56% and 88%, respectively, suggesting the test’s superior ability for ruling out patients without bladder cancer, and an inferior ability for predicting bladder cancer in higher-risk patients. Furthermore, the PLR and NLR usually reflect the diagnostic accuracy in clinical practice [37, 38]. The PLR and NLR of the test was 4.36 and 0.51, respectively, which shows that patients with bladder cancer have approximately 4.36 times higher possibility of testing positive compared with subjects without bladder cancer, as well as a 51% chance of an individual having bladder cancer if the test is negative. The performance of the NMP22 BladderChek test in the pooled PLR and NLR did not achieve the requirements of clinical practice, and remains to be modified for clinical confirmation and exclusion purposes. An AUC of 0.83 represents good discrimination ability for diagnosing bladder cancer [39, 40]. Therefore, the pooled results suggest that the NMP22 BladderChek test has good discrimination ability in clinical practice and is a superior method for ruling out patients at higher risk of bladder cancer.

When tumor stage and grade were considered, the sensitivity was low for Ta tumors. However, we found that the sensitivity of the test increased steadily for identifying tumors of increasing stage, i.e., from Ta to T1 and > T2 (13.68%, 29.49%, and 74.03%, respectively). A similar increasing trend was observed for tumor grade, where sensitivity for G1, G2, and G3 was 44.16%, 56.25%, and 74.03, respectively. The pooled results showed better potential for screening > T2 and high-grade bladder cancer. Moreover, the subgroup analysis showed that, compared with the Caucasian populations, the test had better ability to detect bladder cancer in Asian populations. Considering the increasing incidence of bladder cancer in Asian countries, the NMP22 BladderChek test should be promoted for bladder cancer detection and follow-up [41].

The heterogeneity test and meta-regression analysis showed significant heterogeneity across the included studies, and the quality of these studies may be the potential origin of the relatively high heterogeneity. The included trials involved two main types of diagnostic trials, i.e., case–control and cross-section, and the differences in patient enrollment, study design, and data collection between the two types contributed to the high heterogeneity. In an experimental model, Miyake et al. [10] investigated the potential factors influencing the NMP22 BladderChek test, and reported that the absence of significant urinary cellularity in some cases, depending on the lesion characteristics or the timing of sampling, may lead to false negative results. Therefore, these factors should be considered when exploring the potential source of heterogeneity.

We identified some weaknesses in our review. We could not investigate some influencing factors of the NMP22 BladderChek test, such as the proportions of subjects who smoked, as the included studies had limited published data. Second, some studies were only published as meeting abstracts, and ongoing studies may have been overlooked, which may have contributed to the publication bias detected in our study. Third, we excluded five relevant papers published in languages other than English or Chinese during the literature review and study selection, leading to potential heterogeneity. Moreover, the test has limited efficacy for detecting residual tumors before second transurethral resection of bladder cancer, which has no additional benefit when combined with cytology [42]. In the present study, we could not perform exclusive analysis with regard to this issue; therefore, our results should be interpreted with caution.

## MATERIALS AND METHODS

### Search strategy

We searched MEDLINE, Embase, the Cochrane Library, and Web of Science for relevant studies in all languages published from January 1, 1990, to June 1, 2017, with the following terms: (BladderChek OR NMP22BC) AND (Bladder cancer OR urinary bladder neoplasm [MeSH]). We identified additional studies by screening the reference lists.

### Inclusion and exclusion criteria

The aim of the study selection was to identify clinical studies evaluating the performance of the NMP22 BladderChek test using human urine samples. Studies were included in the systematic review and meta-analysis if they met the following inclusion criteria: (1) Clinical study comparing the diagnostic effects of the NMP22 BladderChek test with at least one measurement for patients with high bladder cancer risk; (2) provided sufficient data for constructing the diagnostic four-fold (2 × 2) contingency table; (3) if data or data subsets were used in more than one article, the article with the most detail or the most recent article was chosen; (4) written in English or Chinese; (5) Related studies with unqualified data or that did not provide sufficient data were included only in the systematic review. The exclusion criteria were: (1) Duplicate publication; (2) reviews, case reports, letters to editors; (3) studies in languages other than English or Chinese. Two reviewers (ZJ Wang and HL Que) independently screened the collected citations for relevance and reviewed full-text articles according to the inclusion criteria. Disagreements were resolved by consultation with a third reviewer (M Gu).

### Data extraction and quality assessment

Two reviewers (ZJ Wang and HL Que) extracted the relevant data independently. The retrieved data included: first author; publication year; ethnicity; number of patients; mean age; proportion of male and smoking patients; specific details of index test used; sensitivity and specificity; true positive, false positive, false negative, and true negative results; sensitivity for bladder tumor grade.

We used the Quality Assessment of Diagnostic Accuracy Studies (QUADAS-2) tool [11] to assess methodological quality. The QUADAS-2 scale contains four domains: patient selection, index test, reference standard, and flow and timing. We assessed all domains for potential risk of bias and the first three domains for concerns regarding applicability. Each question was assigned a “yes,” “no,” or “unclear” response when every eligible study was evaluated. A consensus reviewer (M Gu) resolved any disagreement among the data extraction and quality assessment.

### Statistical analysis

Meta-analysis was performed by pooling raw data on sensitivity, specificity, PLR, NLR, and DOR from the eligible studies employing the sROC curve to express the test parameter results. We assumed that sensitivity and specificity would vary across the studies because of differences in study populations and because of sampling errors, indicating that a random effect model should be used to account for inter-study heterogeneity. The DOR is a single indicator of test performance; a higher DOR value indicates better discriminatory test performance [12]. AUC-ROCs are always close to 1 when tests are accurate; by contrast, tests with poor accuracy usually have AUC-ROCs approaching 0.5 [13]. Heterogeneity was assessed quantitatively using the inconsistency index (I^2^), where I^2^ > 50% indicated substantial heterogeneity. We calculated the Spearman correlation coefficient to check the potential threshold effect. We used the Deeks funnel plot asymmetry test in the diagnostic meta-analysis to evaluate publication bias. All statistical analyses were conducted using Meta-Disc Version 1.4 software and STATA 12.0 software [14]. *P*-values < 0.05 were considered statistically significant.

## CONCLUSIONS

The NMP22 BladderChek test has good discrimination ability for detecting bladder cancer and its high-specificity algorithm can be used for early diagnostic detection to rule out patients with higher bladder cancer risk. Moreover, this test has better potential for screening tumors of higher grade and stage, and has better diagnostic performance in Asians.

## SUPPLEMENTARY MATERIALS FIGURES AND TABLE




