# Supplementary material for: Evaluation of the NMP22 BladderChek test for detecting bladder cancer: a systematic review and meta-analysis
Source: Oncotarget. 2017 Oct 23;8(59):100648–56. doi: 10.18632/oncotarget.22065 (PMC5725051; doi:10.18632/oncotarget.22065)
Supplement: Supplementary file 1 [file oncotarget-08-100648-s001.pdf]

# Evaluation of the NMP22 BladderChek test for detecting bladder cancer: a systematic review and meta-analysis

## SUPPLEMENTARY MATERIALS

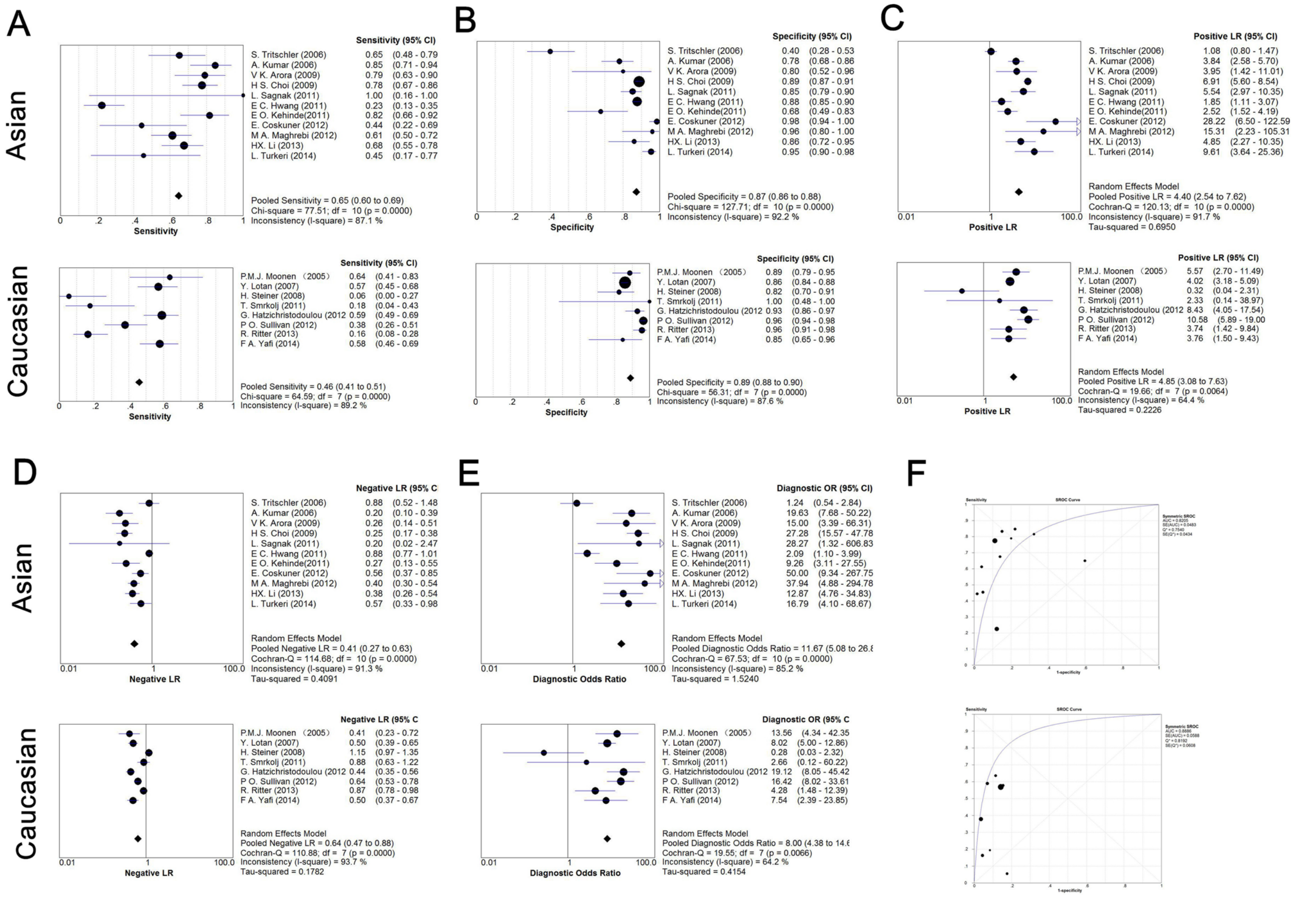


**Supplementary Figure 1:** Meta-analysis of pooled sensitivity (**A**), specificity (**B**), PLR (**C**), NLR (**D**), DOR (**E**), and ROC (**F**) in Asian and Caucasian populations.

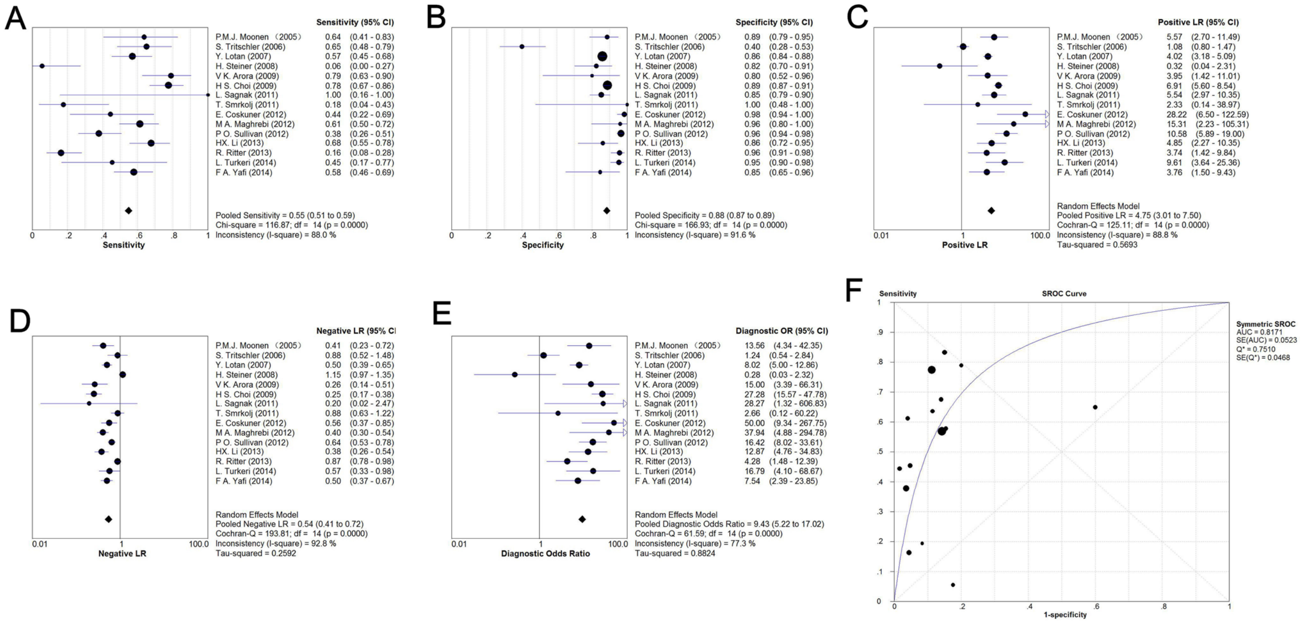


**Supplementary Figure 2:** Meta-analysis of pooled sensitivity (**A**), specificity (**B**), PLR (**C**), NLR (**D**), DOR (**E**), and ROC (**F**) among studies on primary tumor detection.

**Supplementary Table 1: Systematic review of eligible trials in our study.** See Supplementary_Table_1.
