# Supplementary material for: Evaluation of the NMP22 BladderChek test for detecting bladder cancer: a systematic review and meta-analysis
Source: Oncotarget. 2017 Oct 23;8(59):100648–56. doi: 10.18632/oncotarget.22065 (PMC5725051; doi:10.18632/oncotarget.22065)
Supplement: Supplementary file 2 [file oncotarget-08-100648-s002.docx]

**Supplementary Table 1: Systematic review of eligible trials in our study**

| Authors | Ta | | T1 | | ≥T2 | | Tis | | G1 | | G2 | | G3 | | Conclusions |
| --- | --- | --- | --- | --- | --- | --- | --- | --- | --- | --- | --- | --- | --- | --- | --- |
|  | n | Sen (%) | n | Sen (%) | n | Sen (%) | n | Sen (%) | n | Sen (%) | n | Sen (%) | n | Sen (%) |  |
| P.M.J. Moonen （2005） | 15 | 40 | 6 | 83.30 | 7 | 100 | NA | NA | 7 | 28.60 | 9 | 88.90 | 13 | 61.50 | NMP22 BC test has a slightly higher sensitivity compared to cytology, without a relevant loss in specificity. |
| S. Tritschler (2006) | 22 | 55 | 4 | 50 | 3 | 100 | 9 | 78 | 16 | 55 | 26 | | 78 | | NMP22 BC test shows poor specificity and sensitivity and cannot be recommended. |
| A. Kumar (2006) | NA | NA | NA | NA | NA | NA | NA | NA | 11 | 81.8 | 22 | 81.0 | 13 | 92.3 | NMP22 BC test has higher sensitivity for all stages and grades of superficial bladder cancer than cytology. |
| Y. Lotan (2007) | 34 | 47.10 | 29 | 51.7 | 11 | 90.9 | 5 | 80.0 | 34 | 47.1 | 18 | 44.4 | 27 | 77.8 | The PPV of NMP22 BC test improves in patients at higher risk of bladder cancer reaching 77% in men presenting with gross haematuria who are aged >65 years and smoke. The NPV is highest in women aged<65 years, up to 100%. |
| Y. Lotan (2008) | NA | NA | NA | NA | NA | NA | NA | NA | NA | NA | NA | NA | NA | NA | NMP22 BC test for screening an asymptomatic, high risk population can detect noninvasive cancers. |
| H. Steiner (2008) | NA | NA | NA | NA | NA | NA | NA | NA | NA | NA | NA | NA | NA | NA | Although the sensitivity and specificity could not be calculated, the NMP22 BC test added no useful information. |
| V K. Arora (2009) | NA | NA | NA | NA | NA | NA | NA | NA | 25 | 72 | 13 | 92.3 | NA | NA | NMP22 BC test is a useful adjunct to urine cytology in atypical and low-grade carcinoma. |
| H S. Choi (2009) | NA | NA | NA | NA | NA | NA | NA | NA | 56 | 83.9 | 24 | 62.5 | NA | NA | The greater sensitivity of the NMP22 BC test may be more useful for the diagnosis of non-muscle-invasive bladder cancer and low-grade bladder cancer than for the diagnosis of invasive or high-grade bladder cancer. |
| E O. Kehinde(2011) | NA | NA | NA | NA | NA | NA | NA | NA | 25 | 100 | 15 | 100 | 10 | 100 | NMP22 BC test appeared to be most cost-effective and rapid, with relatively high sensitivity and specificity in all categories of patients. |
| T. Smrkolj (2011) | 11 | 9.10 | 3 | 66.7 | 1 | 0 | NA | NA | NA | NA | NA | NA | NA | NA | The NMP22 BC test showed higher sensitivity and lower specificity compared with voided urine cytology, whereas the sensitivity of the NMP22 BC test was low. |
| E C. Hwang (2011) | 59 | 18.64 | 34 | 41.18 | 13 | 61.54 | 1 | 0 | 4 | 0 | 53 | 22.6 | 50 | 42.0 | Combined with cytology, the sensitivity for detecting urothelial carcinoma is increased and may be useful in the screening and follow-up of urothelial carcinoma. |
| L. Sagnak (2011) | NA | NA | NA | NA | NA | NA | NA | NA | NA | NA | NA | NA | NA | NA | NMP22BC test, non-invasive and cost-effective method, for lower tract investigation instead of urine cytology could be recommended. |
| M A. Maghrebi (2012) | 6 | 10 | 53 | 58.5 | 15 | 66.7 | 6 | 23.3 | 38 | 55.3 | 23 | 74 | 19 | 58.0 | NMP22 BC test correlates with tumor pathological grade but not stage. |
| G. Hatzichristodoulou (2012) | 62 | 44 | 18 | 83 | 16 | 100 | 4 | 25 | 37 | 27 | 34 | 77 | 29 | 79 | NMP22BC test may be clinically more useful for bladder cancer detection, especially for high-risk screening. |
| G. Ludecke（2012) | NA | NA | NA | NA | NA | NA | NA | NA | NA | NA | NA | NA | NA | NA | NMP22 BC test are not sensitive for the primary diagnosis of bladder carcinoma. |
| E. Coskuner (2012) | NA | NA | NA | NA | NA | NA | NA | NA | NA | NA | NA | NA | NA | NA | NMP22 BC test is also not reliable in upper urinary tract transitional cell carcinomas, even in high-grade tumors. |
| P O. Sullivan (2012) | 37 | 38 | 16 | 50 | 11 | 27.3 | 2 | 0 | 3 | 33.0 | 38 | 34.0 | 24 | 46 | At the prespecified specificity of 85%, uRNA and Cxbladder-D showed an overall sensitivity that was better than cytology and NMP22 BC test. |
| HX. Li (2013) | NA | NA | NA | NA | NA | NA | 2 | 50.0 | 29 | 51.7 | 15 | 66.7 | 12 | 91.7 | The combination of LBC and NMP22 BC test yielded a similar sensitivity to FISH alone with a slight decrease in specificity. |
| R. Ritter (2013) | NA | 7.3 | NA | 42.9 | NA | 33.3 | NA | NA | 43 | | 5.1 | | 15 | 36.4 | The performance of NMP22 BC test was worse compared to prior studies. |
| F A. Yafi (2014) | NA | NA | NA | NA | NA | NA | NA | NA | NA | NA | 60 | 25 | 23 | 92 | NMP22 BC test was not recommended compared to other novel assays. |
| L. Turkeri (2014) | NA | NA | NA | NA | NA | NA | NA | NA | NA | NA | NA | NA | NA | NA | Combined utilization of RisikoCheck© questionnaire and NMP22 BC test may identify the population with a higher risk of bladder cancer. |
| M D. Bell （2016） | NA | NA | NA | NA | NA | NA | NA | NA | NA | NA | NA | NA | NA | NA | NMP22 BC test was not independently prognostic, which required further validation. |
| Y. Lotan (2017) | 675 | 5.0 | 193 | | 11 | | 101 | 31 | NA | NA | NA | NA | NA | NA | The sensitivity of Cxbladder Monitor significantly outperformed cytology and NMP22 BC test. |

Abbreviations: NMP22 BC test, nuclear matrix protein-22 BladderChek© test; PPV, positive prognostic value; NPV, negative prognostic value; Sen, sensitivity; NA, not available; LBC, liquid-based cytology; FISH, fluorescence in situ hybridization.
